# The effect of herd immunity thresholds on willingness to vaccinate

**DOI:** 10.1057/s41599-022-01257-7

**Published:** 2022-07-18

**Authors:** Per A. Andersson, Gustav Tinghög, Daniel Västfjäll

**Affiliations:** 1 Department of Behavioural Sciences and Learning, Linkoping university, Linkoping, Sweden; 2 Department of Management and Engineering, Linkoping university, Linkoping, Sweden; 3 Department of Health, Medicine and Caring Sciences, Linkoping university, Linkoping, Sweden; 4 Decision Research, Eugene, OR USA

**Keywords:** Psychology, Cultural and media studies

## Abstract

Throughout the COVID-19 pandemic, media and policymakers openly speculated about the number of immune citizens needed to reach a herd immunity threshold. What are the effects of such numerical goals on the willingness to vaccinate? In a large representative sample (*N* = 1540) of unvaccinated Swedish citizens, we find that giving a low (60%) compared to a high (90%) threshold has direct effects on beliefs about reaching herd immunity and beliefs about how many others that will get vaccinated. Presenting the high threshold makes people believe that herd immunity is harder to reach (on average about half a step on a seven-point scale), compared to the low threshold. Yet at the same time, people also believe that a higher number of the population will get vaccinated (on average about 3.3% more of the population). Since these beliefs affect willingness to vaccinate in opposite directions, some individuals are encouraged and others discouraged depending on the threshold presented. Specifically, in mediation analysis, the high threshold indirectly increases vaccination willingness through the belief that many others will get vaccinated (*B* = 0.027, *p* = 0.003). At the same time, the high threshold also decreases vaccination willingness through the belief that the threshold goal is less attainable (*B* = −0.053, *p* < 0.001) compared to the low threshold condition. This has consequences for ongoing COVID-19 vaccination and future vaccination campaigns. One message may not fit all, as different groups can be encouraged or discouraged from vaccination.

## Introduction

On March 11, 2020 the World Health Organization declared COVID-19 a pandemic, calling on countries to take urgent and aggressive actions to protect global health (World Health Organization, 2020). Soon after, the race towards effective vaccines was underway, leading to months of vaccine trials followed by large-scale vaccination campaigns. While some countries managed to reach high vaccination rates among those offered vaccines, other countries were slower in their progress. The success of these campaigns, and future campaigns, will be decided by multiple factors. A key factor is undoubtedly the willingness of people to get vaccinated and effective communication to counteract unwillingness to vaccinate.

Unwillingness to be vaccinated against COVID-19 can stem from a number of sources (Geiger et al., 2021), including fear of negative side effects related to an expedited approval process (Lin et al., 2021), conspiracy theories (Rieger, 2021), as well as not being at risk and having doubts about vaccine efficacy (Goldman et al., 2020). Gallup poll estimates placed the worldwide unwillingness to take the COVID-19 vaccine around 32%, or 1.3 billion people, in May 2021 (Ray, 2021). Willingness to vaccinate is, however, not a fixed trait, and can be affected by information (e.g. Loomba et al., 2021; Roozenbeek et al., 2020) and behavioural interventions in the form of nudges (Dai et al., 2021). For instance, sending unvaccinated people reminder texts that they could be vaccinated and including links to appointment booking increased vaccination rates in a field experiment (Dai et al., 2021). Presenting new incentives to get vaccinated can also increase vaccinations, as in the case of providing small monetary rewards (Campos-Mercade et al., 2021). Similarly, presenting prosocial motives for vaccinations like protecting others from the disease can also increase vaccine willingness (Rieger, 2020).

While individuals themselves can benefit directly from them having been vaccinated, indirect benefits also extend to those who for any reason cannot or will not get vaccinated. This so-called herd immunity can be achieved if enough people reach immunity to stop the spread of a disease, as these immune people act as barriers between the non-immune individuals that could spread the disease. Thresholds for reaching herd immunity depend heavily on the basic reproduction number of a disease (Fine et al., 2011). During the first months of the COVID-19 pandemic, it was estimated that its reproduction number would be in the range of 2–3, placing the threshold of immune individuals needed between 50% and 67% (Omer et al., 2020). In the later stages of vaccine campaigns, several declarations of reaching such thresholds were proclaimed (e.g. McPhillips, 2021; Schraer, 2021; Skopeliti, 2021). However, higher estimates of the threshold for herd immunity were also being discussed due to more transmissible mutations of COVID-19, with experts citing numbers in the 80–90% range (McNamara, 2021). In May 2021 Dr. Anthony Fauci, chief medical advisor to the U.S. President, proclaimed that people were getting confused about these herd immunity thresholds, and noted that he stopped using them in communication because of this (Mandavilli, 2021). Indeed, a number of factors beyond disease reproduction numbers also affect the potential for herd immunity.

Highly important to the COVID-19 pandemic, vaccine effectiveness impacts the potential for reaching herd immunity. The number of vaccinated individuals who were still contracting the disease, called breakthrough cases, was still expected to be “a very small part” of the vaccinated individuals the weeks prior to the present study (Folkhälsomyndigheten, 2021a). However, unlike historical examples like the smallpox vaccine, the COVID-19 vaccines have so far been shown to not provide full immunity, and breakthrough cases became a growing concern in 2021 and onwards (Bergwerk et al., 2021; Dzinamarira et al., 2022). Further, the proportion of vaccinated individuals in the population required to reach a degree of herd immunity will depend on unknown local factors like population mobility and the virus variant circulating, making it more realistic to speak of a spectrum rather than a threshold (Kwok et al., 2021). While flat percentage numbers may be gross simplifications of the dynamics of real spread, these thresholds, which continued to be speculated about in media, may still have a huge impact when communicated. Thus, the aim of the present article was to examine the potential impact of communicating these thresholds on vaccination willingness and related beliefs.

Prior to the COVID-19 pandemic, about a third of US participants in studies had no knowledge about herd immunity (Griffith et al., 2020; Logan et al., 2018). Studies have indicated that such knowledge can affect willingness to vaccinate in general. For instance, knowing that other vulnerable people could benefit from one’s own vaccination can increase willingness to vaccinate (Rieger, 2020; Vietri et al., 2012), and communicating the concept of herd immunity can improve willingness to vaccinate (Betsch et al., 2013, 2017). Communicating the concept of herd immunity using visualizations may be especially effective (e.g. Betsch and Bohm, 2018; Korn et al., 2021). Although there was mixed evidence in relation to parents' willingness to vaccinate children (e.g. Hendrix et al., 2014), and in comparing population benefits to family benefits (Rabb et al., 2021), available studies suggested potential increases in vaccination willingness by communicating population-level benefits (Hakim et al., 2019). Such increases in vaccination willingness were seen as especially likely when the concept of herd immunity could be communicated more successfully, such as by using animations (Korn et al., 2021). During the COVID-19 pandemic, recent studies indicate that knowledge about herd immunity (Pfattheicher et al., 2022), or indirectly protecting others (Rieger, 2020), could positively relate to a willingness to get vaccinated (for an overview see Bohm and Betsch 2022). However, such studies have not mentioned thresholds or percentage goals, which were commonly mentioned in media when discussing COVID-19 and herd immunity.

In general, psychological research shows that setting goals can be central for motivation and performance (Lunenburg, 2011). Communicating high versus low thresholds for herd immunity may act as both motivating and demotivating goals for the individual. While lower thresholds may be perceived as goals that are easier and more realistic to attain, the perceived need for the individual to participate may not be as great, and an easy goal may be less motivating (Locke and Latham, 2006). For higher thresholds, while implying the need for everyone the vaccinate, feelings of inefficacy and “drop in the bucket thinking” may demotivate people (Västfjäll et al., 2015). Further, goals that seem too difficult may also be outright rejected for appearing unattainable (Lunenburg, 2011). There could thus be a paradox at hand, where a higher threshold for herd immunity requires more people to get vaccinated, but also acts as a psychological deterrent, thereby lowering the actual amount of people getting vaccinated.

## Method

### Participants and procedure

A nationally representative sample of 1540 participants was recruited using the panel recruitment company PFM Research (www.pfmresearch.se). These participants were recruited in the time period May 18 to May 27, 2021. At this time, most regions in Sweden were at the start of “Phase 4” of a countrywide vaccination program. This meant that vaccinations were mainly offered to people in risk groups or above the age of 55, with the majority of people below this age unable to even book a time slot for vaccination. To find unvaccinated participants, we recruited only from ages 18 to 51. Any participant who stated they had either been vaccinated or already booked a time for vaccination were screened out before starting the study. After dropping participants failing the attention check (265 participants), being overage (3 participants), or having incomplete data (3 participants), we base our analyses on 1269 (52.8% female, mean age = 36.0) participants in three randomized experimental conditions.

Participants were randomized into one of three conditions: Low herd immunity threshold (LHIT60%), High herd immunity threshold (HHIT90%), or Control (no info about herd immunity). Participants in the two experimental conditions (LHIT60% and HHIT90%) read a short description of herd immunity, which was adapted from the Public Health Agency of Sweden website (Folkhälsomyndigheten, 2021b). This included information stating that herd immunity could protect those people who are unable to vaccinate themselves, and that “Researchers calculate that we can reach herd immunity in Sweden if 60% (90%) of those offered vaccination take the COVID-19 vaccine. This would mean that the disease would stop spreading within the country.” In order to illustrate the effect of herd immunity, an animated GIF image adapted from National Public Radio (2021), showing spread within a low immunity population versus a high immunity population, was also included in the two experimental conditions. Participants in the control condition did not view any information pertaining to herd immunity. Following the end of the survey, participants were fully debriefed. All participants were paid a flat fee of 33 SEK (around 4 USD) for taking part in the survey.

### Measurements

As the main outcome variable participants stated their willingness to vaccinate themselves the next week if they could, which we refer to as Vaccination Now (from 1 = would definitely not get vaccinated, to 7 = would definitely get vaccinated). To explore willingness for consecutive vaccination to prevent the spread of COVID-19 mutations, participants also stated willingness to get vaccinated every 6 months, which we refer to as Vaccination Biannual. Two belief measurements then followed, first an open question regarding how many percent of the population participants believed would get vaccinated (Vac%Pop), then a second belief measure about it being likely to reach the threshold for herd immunity in Sweden given the threshold presented earlier (ReachThresh), where participants answered on a seven-point scale from completely unlikely to completely likely. More beliefs and individual difference questions followed in a separate block of questions, including the beliefs regarding how many close others will get vaccinated (Vac%Close), the attention check, and beliefs that vaccinating oneself would make a difference in the spread of COVID-19 (efficacy). We also asked about risk group status, having had COVID-19, being an urban citizen or not, and demographic questions.

### Statistical analysis

For our mediation analyses, we use bootstrapping with 5000 random samples with replacement, in accordance with Hayes (2009). However, it can be noted that both mediators also produce indirect effects with confidence intervals not crossing the zero point even without bootstrapping. While we do not find a direct effect of the high versus low threshold condition on our outcome vaccination willingness, modern mediation analyses do not require this as a condition, especially when the two mediators produce a positive and a negative indirect effect on the outcome (e.g. Hayes, 2009; Shrout and Bolger, 2002). For transparency we also include the separate mediation models, using only one mediator at a time in supplementary materials Tables S2 and S3. Taken together these separate models show the same type of effects, at comparable sizes, yielding the same interpretation.

## Results

**Fig. 1:**
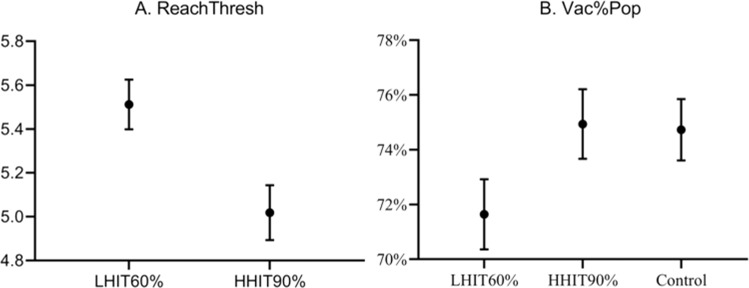
Differences in means between high and low herd immunity thresholds, showing 95% confidence intervals. Note: For **A**, the *Y*-axis is the belief about reaching the threshold (1 = completely unlikely, 4 = either or, 7 = completely likely). For **B**, the *Y*-axis is the percentage one believes will get vaccinated in the total population.

To begin with, Table 1 shows the direct consequences of presenting the different herd immunity thresholds. Presenting herd immunity thresholds did not have a direct effect on willingness to vaccinate, neither for vaccination now nor for biannual vaccination. For the belief that the threshold is attainable (ReachThresh), we unsurprisingly see that participants view the low threshold as significantly more likely to attain. For the beliefs regarding how many close others will get vaccinated (Vac%Close), there was no difference between conditions. For the beliefs regarding how many of the population will get vaccinated (Vac%Pop), we see an increase in this belief from 71.6% in the low threshold to 74.9% in the high threshold, with the control condition similar to the high threshold. Figure 1 illustrates these differences between conditions with confidence intervals.

**Table 1:** Mean values and differences across experimental conditions.

	Low threshold Mean (SD)	High threshold Mean (SD)	Control Mean (SD)	*χ*^2^	df	*p*
Willingness-to-vaccinate: Now	5.91 (1.88)	5.79 (1.93)	5.81 (1.89)	1.423	2	0.491
Willingness-to-vaccinate: Biannual	5.19 (1.93)	5.09 (1.94)	5.23 (1.81)	0.920	2	0.631
ReachThresh	5.50 (1.20)	5.02 (1.32)	.	36.994	1	<0.001
Vac%Pop	71.6 (13.3)	74.9 (13.3)	74.7 (11.6)	21.644	2	<0.001
Vac%Close	85.6 (19.5)	85.6 (20.9)	86.4 (19.1)	0.120	2	0.942
Efficacy	9.13 (1.09)	9.05 (1.08)	9.11 (1.04)	1.847	2	0.397

Differences between conditions were tested using the nonparametric test Kruskal–Wallis.

Noting that the beliefs ReachThresh and Vac%Pop were significantly different between conditions, this was an indication of potential mediating effects. Looking at ReachThresh and Vac%Pop together as potential mediators of vaccine willingness, their different relations with the high and low threshold conditions were indications that there could be both a positive and a negative indirect effect relating to a willingness to vaccinate. From here onwards, we focus on this relation and on comparing the high and low thresholds of herd immunity.

In order to investigate willingness to vaccinate further, we present ordinary least squares (OLS) regression models with control variables. Table 2 shows our regression over willingness to vaccinate for the COVID Vaccination Now scenario. As in the previous analysis, there is no main effect of the condition. Holding a stronger belief that the herd immunity threshold could be attained had a positive effect on increased willingness to vaccinate in all models. The belief that the herd immunity threshold can be attained and the belief that many others will get vaccinated are both significant predictors of willingness to vaccinate in model 3. Comparing 95% CIs for standardized coefficients in model 3, we find ReachThresh [0.180, 0.315] and Vac%Pop [0.169, 0.302] comparable in terms of effects. The coefficients for Model 3 could be interpreted as moving three steps in ReachThresh (e.g. from “Somewhat unlikely” to “Likely” to reach the threshold) or 30 steps (e.g. from the belief that 40% will get vaccinated to 70%) in Vac%Pop, results in moving one step in vaccine willingness (e.g. from “Would likely get vaccinated” to “Would definitely get vaccinated”).

**Table 2:** OLS regression on vaccine willingness.

	Model 1	Model 2	Model 3	Model 4
High threshold condition	−0.149 (0.132)	0.094 (0.128)	−0.077 (0.127)	−0.06 (0.111)
Age	−0.009 (0.007)	−0.009 (0.007)	−0.013^ (0.007)	−0.012* (0.006)
Female	−0.167 (0.132)	−0.108 (0.126)	−0.177 (0.123)	−0.118 (0.108)
Had COVID	−0.027 (0.15)	−0.024 (0.143)	0.005 (0.139)	0.081 (0.122)
Risk group		−0.191 (0.161)	−0.108 (0.157)	−0.082 (0.138)
ReachThresh		0.463*** ((0.05)	0.367*** (0.051)	0.186*** (0.046)
Vac%Pop			0.033*** (0.005)	0.002 (0.005)
Vac%Close				0.05*** (0.003)
Constant	6.577*** (0.349)	3.913*** (0.441)	2.289*** (0.489)	1.096* (0.435)
*n*	829	829	829	829
Adjusted *R*^2^	0.002	0.094	0.143	0.341

All regressions are OLS for willingness to vaccinate the next week. The dependent variable is the willingness to vaccinate the next week (coded from 1 = Would definitely not get vaccinated, through 4 = Either or, to 7 = Would definitely get vaccinated). “Age” is the participant's age in years. “Female” is a dummy for gender (1 = female, 0 = male). “Had COVID” is a dummy for believing one has had COVID-19 (1 = Yes, 0 = No). “Risk group” is a dummy for being in a risk group oneself or sharing household with a risk group person (1 = risk group, 0 = not). “ReachThresh” is the belief that the herd immunity threshold can be achieved (1 = completely unlikely, 7 = completely likely). “Vac%Pop” is the percentage of the population one believes will get vaccinated. “Vac%Close” is the percentage of close others one believes will get vaccinated.

^*p* < 0.10, *p < 0.05, ***p < 0.001.

In short, this means that a stronger belief that the vaccine threshold can be reached and a belief that more people will get vaccinated both increases willingness to vaccinate. However, adding the belief about how many close others will get vaccinated (Vac%Close) reduces the relation between the beliefs about the population (Vac%Pop) and vaccine willingness in model 4. Instead, we see that the belief that close others will get vaccinated is a very strong predictor, adding much-explained variance in model 4. It should be noted that multicolinearity within the model is still low (VIF values are all below 1.5) and the two beliefs regarding how many close others and how many in the population will get vaccinated are not highly correlated (*r* = 0.480). Still, it would be reasonable to assume that part of the effect of the belief that others will get vaccinated is contained in the belief that close others will get vaccinated, as it is a subgroup of the larger group, and also a particularly influential subgroup. Looking at other variables, having had COVID and being in a risk group does not seem to strongly predict willingness to vaccinate. However, the sample does not include the oldest cohorts, who should be most at risk.

**Fig. 2:**
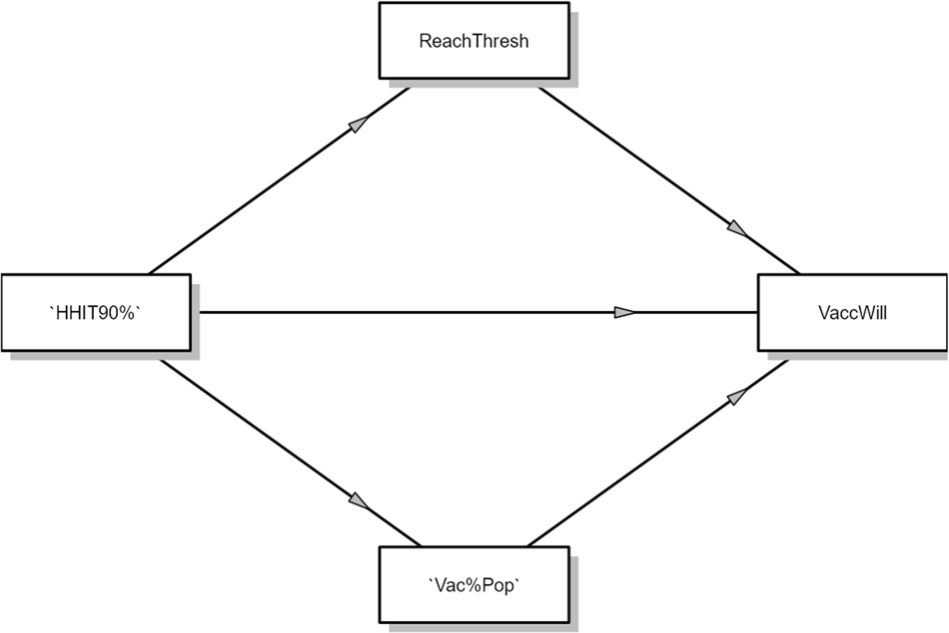
Mediation model diagram. The mediation model puts the two beliefs, that the herd immunity threshold can be reached (ReachThresh) and that others in the population will get vaccinated (Vac%Pop), as mediators between the high herd immunity threshold condition and vaccine willingness.

To test if the beliefs about attaining the herd immunity threshold and the belief that others in the population will get vaccinated act as mediators between our manipulation and vaccine willingness we perform a mediation analysis. This mediation is modelled as shown in Fig. 2, where the two belief variables are placed as mediators, affecting vaccine willingness by the manipulation of high and low herd immunity thresholds. Table 3 shows the full results of the mediation analysis. Both of the beliefs, about reaching the threshold and about the number of the population getting vaccinated, have an effect on the willingness to vaccinate and are affected by the herd immunity thresholds. We find a negative indirect effect at 95% CI [−0.292, −0.118], where the high threshold (HHIT90%) decreases the belief that the threshold can be attainted, which in turn decreases willingness to vaccinate. For the belief that the population will get vaccinated (Vac%Pop) we find a positive indirect effect at 95% CI [0.040, 0.175], as participants in the high threshold condition believe more people will get vaccinated, which in turn increases vaccine willingness. These two indirect effects thus have opposite effects on vaccine willingness, and due to being comparable in size they cancel each other out in terms of an overall effect of presenting a high or low threshold.

**Table 3:** Results of mediation analysis of the effects of two beliefs on the willingness to vaccinate now, through the manipulation of presenting the high or the low threshold as goals.

Type	Effect	Estimate	SE	Lower CI	Upper CI	*β*	*z*	*p*
Indirect	HHIT90% ⇒ ReachThresh ⇒ VaccWill	−0.199	0.045	−0.292	−0.118	−0.053	−4.426	<0.001
Indirect	HHIT90% ⇒ Vac%Pop ⇒ VaccWill	0.102	0.035	0.040	0.175	0.027	2.942	0.003
Component	HHIT90% ⇒ ReachThresh	−0.503	0.086	−0.675	−0.337	−0.196	−5.825	<0.001
Component	ReachThresh ⇒ VaccWill	0.396	0.065	0.263	0.518	0.271	6.106	<0.001
Component	HHIT90% ⇒ Vac%Pop	3.294	0.916	1.442	5.101	0.123	3.598	<0.001
Component	Vac%Pop ⇒ VaccWill	0.031	0.006	0.020	0.043	0.222	5.229	<0.001
Direct	HHIT90% ⇒ VaccWill	−0.034	0.131	−0.296	0.223	−0.009	−0.260	0.795
Total	HHIT90% ⇒ VaccWill	−0.131	0.131	−0.388	0.126	−0.035	−1.001	0.317

HHIT90% refers to the 90% herd immunity threshold (dummy coded as 1 = the high 90% condition, 0 = the low 60% condition). Confidence intervals computed with the method bootstrap percentiles. Betas are completely standardized effect sizes.

In relation to willingness to get vaccinated biannually, we see an identical pattern of indirect effects, with comparable effect sizes. See Supplementary Table S1 for details.

## Discussion

As the percentage threshold for reaching herd immunity has been heavily discussed in media and set as an aim by authorities (e.g. Powell, 2020) we set out to assess the impact of this type of information on the general public. Using a representative sample of Swedish citizens under the age of 51, that were not yet vaccinated against COVID-19, we found support for indirect effects on vaccine willingness through changed beliefs. People who were presented with the high threshold of a 90% vaccination rate needed for herd immunity were less inclined to believe that the threshold was attainable, a belief which itself had a negative effect on vaccination willingness. At the same time, people who were presented with this high threshold also believed that more other people in the population would get vaccinated, a belief that had a positive effect on vaccination willingness. These indirect effects mean that different groups of people may be encouraged or discouraged from vaccination when similar herd immunity thresholds are discussed. Although presenting the concept of herd immunity by itself has previously been seen to have positive effects on vaccine willingness (Betsch et al., 2013, 2017; Korn et al., 2021), presenting the concept along with thresholds provides a different context, as seen here. Our results add to this literature by showing that when it comes to communication of vaccine goals like herd immunity thresholds one message does not fit all.

In terms of limitations, it should be noted that corporate media and government authorities are not the only sources of information that affect behaviour. Information and opinions about vaccines and disease spread in complex networks, which include social networks and interactions between individuals. Following examples such as Yin et al. (2022), future research should continue to model how vaccine-related information spread in such systems. It should also be mentioned that during the later part of 2021 it became more apparent that the current vaccines would not provide full immunity to COVID-19 (Bergwerk et al., 2021), which in turn affected the potential for herd immunity.

Communicating that a nation needs to reach a high threshold for herd immunity, in this case, a 90% vaccination rate for those offered the vaccine, could have mixed effects on the population. The paradox at hand is that while a higher threshold implies a stronger need for everyone to get vaccinated, it may also be discouraging due to it being harder to achieve. The threshold of immunization needed to reach herd immunity will continue to be discussed, in terms of ranges or absolute numbers, and due to mutations such as the delta variant (B.1.617.2) having a higher reproduction number (Campbell et al., 2021), we can expect a clear upward shift in this threshold over time. At the moment of writing, this shift can be traced from initial WHO estimates of around 60% upwards through speculations of about 70–85% (McPhillips, 2021) to later discussion of around 90% of the population (McNamara, 2021). Further, the realities of continued vaccine resistance in pockets of populations who remain resolute in their decision (e.g. Laughlin and Shelburne, 2021), and the existence of a portion of the population who cannot take the vaccine due to risk factors, may lead to such high goals proving hard or even impossible to reach. Finally, models of herd immunity rely on people becoming immune to the point of not transmitting disease, which may be unrealistic when dealing with COVID-19 (Aschwanden, 2021), and waning immunity may lead to the need for both booster shots and global vaccination programs (Dzinamarira et al., 2022). A cautionary approach to communicating these thresholds and the goal of herd immunity could thus be advisable. Rather than the focus on a high threshold needed for complete herd immunity, it may be more realistic to disseminate the notion of gradual herd immunity, focusing on the positive gradual gains towards increased immunity in society. The fight against COVID-19 should perhaps not be communicated as a sprint towards a threshold but as an endurance race.

## Supplementary information

Supplementary materials

## Data Availability

The dataset analysed in the current study are available in the Open Science Framework repository, https://osf.io/7nbzr/?view_only=1b1483f5ce494d809f08eaa529bed982.
