# Supplementary material for: The effect of herd immunity thresholds on willingness to vaccinate
Source: Humanit Soc Sci Commun. 2022 Jul 18;9(1):243. doi: 10.1057/s41599-022-01257-7 (PMC9294790; doi:10.1057/s41599-022-01257-7)
Supplement: Supplementary file 1 — Supplementary materials [file 41599_2022_1257_MOESM1_ESM.docx]

**Supplementary materials**

**The effect of herd immunity thresholds on willingness to vaccinate**

Per A. Andersson, Gustav Tinghög, Daniel Västfjäll

Corresponding author: Per A. Andersson

Corresponding author email: per.a.andersson@liu.se

Supplementary Table S1. Results of mediation analysis of the effects of two beliefs on the willingness to vaccinate biannualy, through the manipulation of presenting the high or the low threshold as goals.

| Type | Effect | Estimate | SE | Lower C.I. | Upper C.I. | β | z | p |
| --- | --- | --- | --- | --- | --- | --- | --- | --- |
| Indirect | HHIT90% ⇒ ReachThresh ⇒ VaccWill | -0.192 | 0.041 | -0.279 | -0.119 | -0.050 | -4.687 | < .001 |
| Indirect | HHIT90% ⇒ Vac%Pop ⇒ VaccWill | 0.105 | 0.034 | 0.045 | 0.178 | 0.028 | 3.052 | 0.002 |
| Component | HHIT90% ⇒ ReachThresh | -0.503 | 0.085 | -0.670 | -0.339 | -0.196 | -5.907 | < .001 |
| Component | ReachThresh ⇒ VaccWill | 0.382 | 0.059 | 0.264 | 0.493 | 0.257 | 6.441 | < .001 |
| Component | HHIT90% ⇒ Vac%Pop | 3.294 | 0.907 | 1.518 | 5.091 | 0.123 | 3.632 | < .001 |
| Component | Vac%Pop ⇒ VaccWill | 0.032 | 0.006 | 0.021 | 0.044 | 0.224 | 5.488 | < .001 |
| Direct | HHIT90% ⇒ VaccWill | -0.023 | 0.134 | -0.287 | 0.237 | -0.006 | -0.169 | 0.866 |
| Total | HHIT90% ⇒ VaccWill | -0.110 | 0.134 | -0.372 | 0.152 | -0.028 | -0.822 | 0.411 |

Note: HHIT90% refers to the 90% herd immunity threshold (dummy coded as 1=90% condition, 0=60% condition). Confidence intervals computed with method bootstrap percentiles. Betas are completely standardized effect sizes.

Supplementary Table S2. Results of mediation analysis of the effect of the belief about reaching the threshold on vaccine willingness now, through the manipulation of presenting the high or the low threshold as goals.

| Type | Effect | Estimate | SE | Lower C.I. | Upper C.I. | β | z | p |
| --- | --- | --- | --- | --- | --- | --- | --- | --- |
| Indirect | HHIT90% ⇒ ReachThresh ⇒ VaccWill | -0.227 | 0.046 | -0.322 | -0.142 | -0.059 | -4.914 | < .001 |
| Component | HHIT90% ⇒ ReachThresh | -0.493 | 0.086 | -0.659 | -0.326 | -0.193 | -5.767 | < .001 |
| Component | ReachThresh ⇒ VaccWill | 0.459 | 0.057 | 0.343 | 0.567 | 0.303 | 8.036 | < .001 |
| Direct | HHIT90% ⇒ VaccWill | 0.125 | 0.134 | -0.137 | 0.391 | 0.032 | 0.931 | 0.352 |
| Total | HHIT90% ⇒ VaccWill | -0.101 | 0.133 | -0.362 | 0.159 | -0.026 | -0.762 | 0.446 |

Note: HHIT90% refers to the 90% herd immunity threshold (dummy coded as 1=90% condition, 0=60% condition). Confidence intervals computed with method bootstrap percentiles. Betas are completely standardized effect sizes.

Supplementary Table S3. Results of mediation analysis of the effect of the belief about how many in the population will get vaccinated on vaccine willingness now, through the manipulation of presenting the high or the low threshold as goals.

| Type | Effect | Estimate | SE | Lower C.I. | Upper C.I. | β | z | p |
| --- | --- | --- | --- | --- | --- | --- | --- | --- |
| Indirect | HHIT90% ⇒ Vac%Pop ⇒ VaccWill | 0.136 | 0.043 | 0.057 | 0.224 | 0.035 | 3.176 | 0.001 |
| Component | HHIT90% ⇒ Vac%Pop | 3.294 | 0.924 | 1.475 | 5.052 | 0.123 | 3.564 | < .001 |
| Component | Vac%Pop ⇒ VaccWill | 0.041 | 0.006 | 0.030 | 0.053 | 0.286 | 7.130 | < .001 |
| Direct | HHIT90% ⇒ VaccWill | -0.246 | 0.129 | -0.495 | 0.010 | -0.064 | -1.906 | 0.057 |
| Total | HHIT90% ⇒ VaccWill | -0.110 | 0.134 | -0.372 | 0.152 | -0.028 | -0.822 | 0.411 |

Note: HHIT90% refers to the 90% herd immunity threshold (dummy coded as 1=90% condition, 0=60% condition). Confidence intervals computed with method bootstrap percentiles. Betas are completely standardized effect sizes.
